# Effect of sodium dodecyl sulfate on the corrosion protection behaviour of mild steel in acidic medium

**DOI:** 10.1098/rsos.250015

**Published:** 2025-06-11

**Authors:** Chandradip Kumar Yadav, Brahamdeo Yadav, Neelam Shahi, Tulasi Prasad Niraula, Yogesh Chaudhary, Manoj Adhikari, Jyotendra Kunwar, Shova Neupane, Amar Prasad Yadav, Ajaya Bhattarai, Dileep Kumar

**Affiliations:** ^1^ Central Department of Chemistry, Tribhuvan University, Kirtipur, Nepal; ^2^ Amrit Science Campus, Tribhuvan University, Thamel, Kathmandu, Nepal; ^3^ Surynarayan Sataynarayan Morbaita Yadav Multiple Campus, Siraha, Nepal; ^4^ Department of Chemistry, Mahendra Morang Adarsh Multiple Campus, Tribhuvan University, Biratnagar, Nepal; ^5^ Laboratory for Chemical Computation and Modeling, Institute for Computational Science and Artificial Intelligence, Van Lang University, Ho Chi Minh City, Vietnam; ^6^ Faculty of Applied Technology, School of Technology, Van Lang University, Ho Chi Minh City, Vietnam

**Keywords:** corrosion, critical micelle concentration, mild steel, surfactant, Tafel plot

## Abstract

The effectiveness of sodium dodecyl sulfate (SDS) is investigated as an inhibitor against a 0.5 M H_2_SO_4_ solution on mild steel (MS). The method was conducted for the calculation of critical micelle concentration (CMC) calculation, weight loss and potentiodynamic polarization. The SDS-coated steel samples were characterized by field emission scanning electron microscopy, Fourier transform infrared spectroscopy, X-ray energy dispersive spectroscopy and atomic force microscopy to understand the surface morphologies after exposure to the CMC of SDS solution. Conductivity measurements were performed to determine the CMC of the SDS solution. It appears that SDS is a perfect inhibitor of MS corrosion, as the weight loss measurement result found an inhibition efficiency of SDS of approximately 98.37% in 0.5 M H_2_SO_4_ solution. We also found, from the Tafel plot, a corrosion current of 0.98 × 10^−3^ A cm^−2^ at CMC (0.0077 M), when MS was dipped for 9 h in 0.5 M H_2_SO_4_. SDS enhances the corrosion resistance of MS in acidic environments and its potential implications for industrial applications. It varies with SDS concentrations, which is attributed to the development of a protective Fe^2+^–SDS complex on the MS surface.

## 1. Introduction

**Figure 1.**
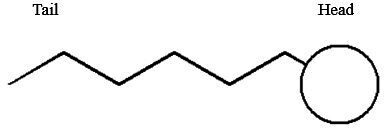
Schematic representation of surfactant monomer.

**Figure 2.**
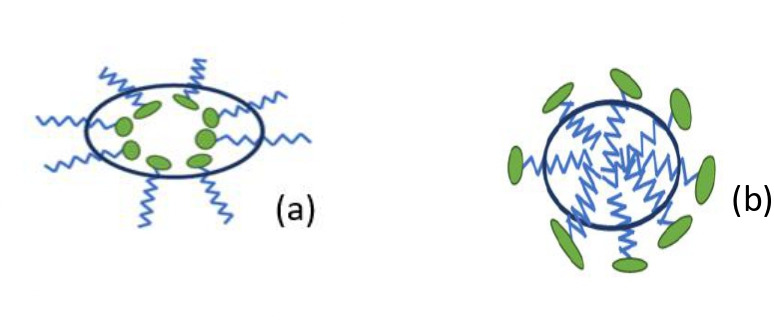
(a) Micelles. (b) Reverse micelles.

Mild steel (MS) is widely used as an engineering material and industry, and its corrosion in acidic environments, such as in the chemical processing, oil, gas and construction industries, has significant economic importance [1]. Most acidic industrial applications, including acid pickling, oil recovery and descaling, utilize steel as a primary material. Acids most commonly used in these processes include sulfuric, hydrochloric, hydrofluoric, nitric, acetic and citric acids. Although MS is passivated in concentrated nitric acid (65%), it dissolves in lower concentrations of the same acid. Numerous organic compounds containing unsaturated bonds and heteroatoms such as nitrogen (N), oxygen (O), sulfur (S) and phosphorus (P) have demonstrated effective corrosion inhibition properties [2]. However, many of these compounds are expensive and highly toxic both to living organisms and to the environment. To address these limitations, the search for renewable, cost-effective, eco-friendly and non-toxic corrosion inhibitors is crucial. Several studies have successfully reported the use of surfactants as effective corrosion inhibitors for MS in acidic media. Surfactants are molecules with a hydrophilic head and hydrophobic tail, which exhibit amphiphilic characteristics (figure 1). Due to having a special architecture, they are widely used in many research objectives [3–7]. When the surfactant is in the solvent, these molecules adsorb onto surfaces, shielding them from aggressive media. In bulk solutions, surfactants can form micelles (figure 2); a process of gathering the surfactant that initiates at a specific concentration is called the critical micelle concentration (CMC) [8–12].

The CMC is an essential parameter that represents the concentration at which surfactant molecules assemble in a solution to form micelles and helps to understand the surfactant behaviour in a solution [13–16]. It governs the transition from monomers to micelles, influencing key features such as surface tension reduction, emulsification and solubilization. The specific CMC value varies according to the type of surfactant and surrounding environmental conditions [17–20]. Surfactant molecules exist as monomers below the CMC; above it, they aggregate into dimers and trimers. The CMC significantly influences the behaviour and effectiveness of surfactants by micelles-structure formations where the hydrophobic tails are shielded from the surrounding solvent (typically water). At the same time, the hydrophilic heads remain exposed [21–24]. Surface tension is reduced above the CMC when micelles form and adhere to surfaces that exhibit the capability to solubilize hydrophobic compounds in their interiors. These features are widely used in numerous applications, including detergents and pharmaceutical formulations [25–28]. Variables such as the amount of electrolytes, pH and temperature influence the CMC, which may affect the surfactant’s ability to form micelles. Common methods for determining CMC are surface tension measurements, conductivity assessments and spectroscopic approaches [29–33].

Surfactants are also used in research to better protect the metal surface [34–39]. The unique role of SDS is as a corrosion inhibitor in acidic media. Surfactants, organic molecules containing elements like nitrogen, oxygen, sulfur, phosphorus and aromatic rings, can bind to metal surfaces. The protection against MS corrosion has been achieved through different methods, such as coating the organic layer, oxidizing treatment and using inhibitory chemicals [40–43]. This adsorption, crucial for corrosion inhibition, forms a protective barrier that blocks active sites and hinders the access of the aggressive medium to the metal, thus slowing down dissolution [44–48]. Within the field of corrosion inhibition, copper can be effectively inhibited by SDS in acidic situations [49]. The SDS also showed its inhibition efficiency in nickel-containing metals in acidic solutions [50]. Furthermore, studies on the impact of SDS inhibition on carbon steel revealed a protective coating composed of Fe^2+^–SDS complexes on the metal surface [51]. This underscores the versatility of SDS as a corrosion inhibitor, demonstrating its efficacy on various metal substrates. MS, a widely used metal in transportation, construction and maritime industries due to its availability, affordability and robust mechanical and thermal properties, is susceptible to corrosion. Traditional organic inhibitors and surfactants mitigate this by forming a protective layer that isolates MS from corrosive agents, particularly crucial in rust-prone marine environments.

In this study, various concentrations of SDS were prepared in 0.5 M H_2_SO_4_ to explore its effects on both above and below CMC [52]. Using 0.5 M H_2_SO_4_ simulates the conditions found in these industries, making the study relevant and applicable to real-world scenarios. Sulfuric acid (H_2_SO_4_) is a strong acid commonly used in industrial processes, and its concentration of 0.5 M provides a sufficiently aggressive environment to test the effectiveness of corrosion inhibitors like SDS. This concentration is strong enough to accelerate corrosion but not so high that it overwhelms the protective effects of the inhibitor. Potentiodynamic polarization and weight loss techniques were used to examine the corrosion behaviour of MS at various SDS concentrations [53]. This research explores the mechanism of SDS corrosion inhibition in MS within a 0.5 M H_2_SO_4_ solution, focusing on reducing the detrimental effects of SDS in the CMC and prolonging the metal’s lifespan. This study evaluates the adsorption and inhibition mechanism of SDS on corrosion. The novel surfactant SDS protects the surface of the MS, which is identified by using weight loss, potentiodynamic techniques, X-ray energy dispersive spectroscopy (EDX), field emission scanning electron microscopy (FESEM), atomic force microscopy (AFM) and Fourier transform infrared spectroscopy (FTIR) investigations on the MS surface. In the investigation, the surface of MS coated with SDS was found.

## 2. Material and methods

### 2.1. Materials

The MS used in the corrosion investigations was cut to 15.92 mm × 13.38 mm × 1.31 mm and purchased in the local market in Kathmandu, Nepal. SiC paper (silicon carbide) in sizes 600, 1000, 1200 and 2000 was used to polish the samples. Subsequently, they underwent a wash with hexane (95% pure), a 25-min ethanol (99.9% purity) sonication and an air blower drying process. The SDS (molecular mass 288.38 g mol^−1^ and product no. 151-21-3 with lot no. 2475741217, 99% purity) was utilized as an inhibitor. It was purchased from Sigma Labsys in Bengaluru, India. SDS solutions in 0.5 M H_2_SO_4_ (AR grade 97% purity) were produced for 0.0019 M, 0.0034 M, 0.0077 M and 0.0154 M. It was purchased from Chemical Drug House in Daryaganj, New Delhi, India, and double-distilled water was used to prepare each solution.

### 2.2. Methods

#### 2.2.1. Critical micelle concentration measurement

The conductivity method is applied along with a conductivity metre purchased from Milwaukee Instruments Pvt. Ltd., Romania, where the CMC of the SDS was determined by the research laboratory of the Central Department of Chemistry, Tribhuvan University, Nepal.

#### 2.2.2. Weight loss method

After being freshly cut and polished, MS specimens were immersed in 25 ml of SDS solution at 298.15 K at the following concentrations: 0.0154 M, 0.0019 M, 0.0034 M and 0.0077 M. The beakers were 50 ml in size. The volume of solution was selected in such a way that the MS plate was fully immersed in the solution. The volume of the acidic solution should be sufficient to fully immerse the MS sample, ensuring that the entire surface is exposed to the corrosive environment. A smaller volume of 25 ml has been chosen if the sample size is small, allowing for complete immersion without wasting excessive amounts of acid. Smaller volumes can facilitate better control over experimental conditions, such as temperature and concentration gradients, which are crucial for obtaining reliable and reproducible results. The starting weight of the sample was subtracted from their weight at a specific interval to determine the weight reduction [54]. A total of 9 h were spent immersing the MS sample in the SDS solution to calculate the weight loss result.

#### 2.2.3. Potentiodynamic measurements

A Gamry instrument (Gamry Instruments, Warminster, PA) was used to measure potentiodynamic polarization via three-electrode systems controlled by AUTOLAB’s GPES software. The working electrode consisted of the MS sample in 25 ml of an acidic solution, with or without the presence of SDS, depending on the specific test conditions. SDS concentrations in 0.5 M H_2_SO_4_ solutions were carefully controlled for this investigation. The set-up consisted of a platinum wire counter electrode, a MS working electrode and a saturated calomel electrode (SCE, +0.241 V) as a reference. Polarization curves were recorded at a scan rate of 0.5 mV s^−1^, scanning from −0.3 V to +0.3 V relative to the open circuit potential (OCP). All measurements were performed at 25°C. Specimens were immersed for 30 min prior to measurement to achieve a stable potential. The inhibition efficiency (IE) was then calculated from the measured corrosion current densities (*I*_corr_) using the following equation:


$$Corrosion\;inhibition\;efficiency=\frac{I_{Corr\;}-I^{*}corr}{I_{Corr}}\times 100$$

where *I*_corr_ = corrosion current in the absence of an inhibitor and I*_corr_ = corrosion current in the presence of an inhibitor.

#### 2.2.4. Characterization techniques

With and without inhibitors, the surface morphology of the corroded MS sample was studied by FTIR (Bruker Company Limited, USA), FESEM (JEOL JSM IT800 field emission scanning electron microscopy, Japan), EDX (Elect Super Company EDAX, USA) and AFM (Bruker JPK Nanowizard, model 02069, Germany).

Before any corrosion response started, the surface morphology of MS was examined by using an optical microscope to search for surface flaws like pits or blatant anomalies like cracks. Samples with a smooth and pet-free surface were immersed. For 9 h at 298.15 K, the samples were dipped in 0.0077 M SDS of 0.5 M H_2_SO_4_. Following the test, the specimens were cleaned in double distilled water, dried and examined with FTIR, FESEM, EDX and AFM.

## 3. Results and discussion

### 3.1. Critical micelle concentration

Conductivity measurements at 298.15 K were used to calculate the CMC of the SDS [55]. The concentration of the SDS surfactant in the solution varied based on the CMC value. This modification was designed to initiate a redox reaction on the surface of MS, with varying amounts of SDS both below and above the CMC [56]. These adjustments were made to facilitate further electrochemical studies.

**Figure 3.**
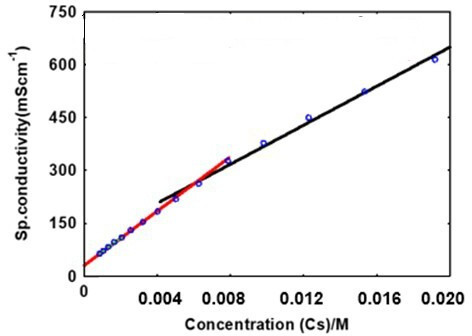
Conductivity of 0.5 M H_2_SO_4_ solution with the presence of SDS at different concentrations. SDS, sodium dodecyl sulfate.


In figure 3, the concentration of surfactant (SDS) causes a linear increase in conductivity. The CMC was calculated from the intersection of lines with varying slopes. The CMC was the surfactant concentration required to saturate the solution and was calculated from acute breaks in the conductivity versus surfactant concentration curve [57]. It is observed that the CMC of SDS is 0.0077 M, obtained by solving the equation.

$${\text{Straight line, y = +3.69E}}^{4}x1+302$$

$${\text{Straight line, y = +8.44E}}^{4}x1+144$$

The conductivity of the 0.5 M H_2_SO_4_ solution increases with the introduction of SDS. This increase is attributed to the higher concentration of SDS ions. The CMC is reached, leading to the formation of micelles. Compared with individual surfactant molecules, the aggregated micelle is significantly larger [58]. However, the larger micelle exhibits mobility lower than that of a single SDS surfactant molecule, resulting in a slower increase in conductivity. This change in pace is evident in the altered slope observed in the concentration and conductivity plots. The CMC for SDS in 0.5 M H_2_SO_4_ is determined to be 0.0077 M [59]. The observed decrease in the CMC of SDS surfactant in an acidic solution is primarily attributed to a reduction in electrostatic repulsion between the anionic hydrophilic head group. This reduction leads to a diminished contribution of repulsion opposing micellization as reported by [60]. Consequently, in an acidic environment, anionic surfactants tend to form micelles more readily, resulting in a lower surfactant CMC in 0.5 M H_2_SO_4_ compared with pure water.

### 3.2. Anticorrosion behaviour of the surfactant

#### 3.2.1. Weight loss measurements

The weight loss approach was used to measure the inhibition efficiency (IE) of SDS. The weight of the MS samples was measured using a four-digit electronic analytical balance Ohaus E1RR80 before and after immersion in 25 ml of 0.5 M H_2_SO_4_ and at different concentrations of 0.5 M H_2_SO_4_-SDS., according to [61]. Evaluation of the inhibitor’s effect included increasing the concentration of surfactant inhibitor to 298.15 K. The effectiveness of the corrosion inhibition process was investigated with various inhibitor concentrations, achieved by immersing MS samples in SDS solutions of 0.0019 M and 0.0034 M. The inhibitor concentration of 0.0077 M, determined using the Easy Plot programme in figure 3, was notably higher, measuring 0.0154 M, exceeding the CMC.

After each measurement, the MS samples were cleaned with double-distilled water, rinsed with acetone, air-dried and then weighed [62]. In compliance with good laboratory practices, each experiment was conducted three times, the results reported as averages. The following common formulas were used to calculate the corrosion rate (CR) and IE:


$$\text{Corrosion rate (CR) = }\frac{\text{Weight loss (W)}}{\text{area (A)×time(T)×density(d)}}\times 8.76\times 10^{4}$$

where *A* is the MS's area (cm^2^), *W* is the weight loss of the MS (g) after immersion time *T* (hours), and *d* is the MS's density (g cm^−3^).


$$\text{Surface Coverage}\;(\theta )=W_{o}–W_{i}/W_{o}$$

$$\text{Inhibition Efficiency (IE)}\%=\left(W_{o}-W_{i}/W_{o}\right)\;\times 100$$

where *W*_o_ represents the weight loss of MS when an inhibitor is not present and *W*_i_ represents the weight loss of MS when an inhibitor is present.

Based on the SDS concentrations, variations in the corrosion rate of MS samples in a 0.5 M H_2_SO_4_ solution can be attributed to the influence of the surfactant SDS on the samples [63]. As the amount of SDS increases, the rate of corrosion reduces (electronic supplementary material, table S1). When the concentration of surfactant increases, the number of surfactant molecules that are adsorbed on the surface of MS increases, which is responsible for the decrease in corrosion rate [64].

Metals typically corrode when exposed to air, moisture, corrosive chemicals or other environmental factors, causing corrosion products to develop on the metal’s surface. Surface coverage, a crucial metric of the corrosion process, indicates the area of the metal surface that is covered with corrosion-related products or by-products [65]. By examining the surface coverage of the corrosion products, one can estimate the direction and magnitude of the corrosion can be estimated. Using the weight loss method, surface coverage varies for 9 h at 0.5 M H_2_SO_4_ with varying concentrations of surfactant (SDS) concentrations [66].

The effectiveness of the corrosion inhibitor in preventing corrosion is shown by the percentage of inhibition efficiency. For example, if the inhibitor has a 90% IE, then only 10% of the corrosion rate would occur if the inhibitor was not there [67]. Several factors influence the inhibition efficiency of a corrosion inhibitor, including the type and concentration of the inhibitor, the parameters of the corrosive environment (e.g. pH, temperature and chemical composition) and the characteristics of the material under protection [68].

**Figure 4.**
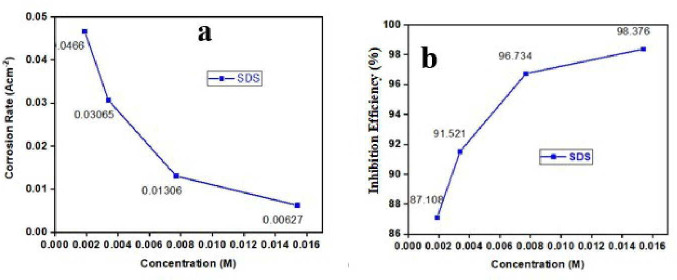
(a) Corrosion rate versus concentration. (b) Inhibition efficiency (%) versus concentration.

As illustrated in figure 4, the IE increases with the increasing concentration of surfactant SDS. Similar patterns were observed in the case of EDTA, an organic compound when the gravimetric technique was applied. These findings suggest that elevating the concentration of surfactants enhances the availability of inhibitor molecules for adsorption onto the MS surface, consequently reducing the surface area susceptible to direct acid attack on the metal surface. Surfactant SDS at concentrations of 0.0019 M inhibits at 87.11%, SDS at 0.0034 M at 91.52%, SDS at 0.0077 M at 96.73% and SDS at 0.0154 M at 98.38%. The surfactant is an effective MS inhibitor. SDS, a surfactant, demonstrated a better efficiency of approximately 98.38%. Water molecules also play a function on the MS sheet. When weighted after immersion in solution, it is reduced but not removed by drying with an air blower [69]. When SDS is directly adsorbing on the surface of MS, it inhibits corrosion. However, in its unsubstituted form, SDS interacts with the metal while a hydration sheath hinders direct contact with the surface. Despite this, its high inhibition efficiency can be attributed to factors such as electric charge, molecular orientation, shape and size.

#### 3.2.2. Potentiodynamic polarization method

Before each polarization experiment, MS samples were submerged in the test solution for 30 min to provide a stable OCP. Electrochemical studies were conducted using a Gamry 600 potentiostat and Gamry framework software (Gamry Instruments, Warminster, PA) [70]. Each measurement was performed using a cylindrical glass cell with three electrodes and 25 ml of test fluid. A polished MS sample with 0.608 cm² of exposed surface area was used as the working electrode. Saturated calomel electrodes (SCEs) as the reference electrode and platinum wires were utilized as the counter electrode. Potential values for SCE are cited in this investigation [71].

To determine the potential from the cathodic to the anodic limits, we recorded potential dynamic polarization at a scan rate of 0.5 mV s^−1^ from −0.3 V to +0.3 V versus OCP. The resulting polarization curves were used to determine cathodic and anodic slopes [72]. For each test solution, the corrosion potential (*E*_corr_) and the corrosion current (*I*_corr_) were estimated using the standard Tafel extrapolation method. The corrosion IE was calculated using the following equation:


$$Corrosion\;inhibition\;efficiency=\frac{I_{Corr\;}-I^{*}corr}{I_{Corr}}\times 100$$

where I*_corr_ is the corrosion current in the presence of an inhibitor and *I*_corr_ is the corrosion current in the absence of an inhibitor.

**Figure 5.**
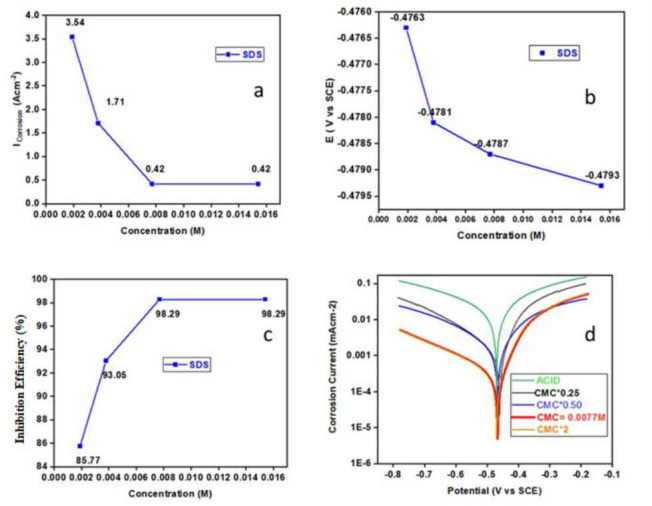
(a) I_corr_ versus concentration. (b) Corrosion potential versus concentration. (c) Inhibition efficiency (%) versus concentration. (d) Corrosion current versus potential.

An inhibitor and the absence of an inhibitor are shown in electronic supplementary material, table S2, corrosion potentials and inhibition efficiency. Figure 5a,d show that when the surfactant level increased, the corrosion rate decreased. The surfactant adsorption is responsible for the decrease in the rate of corrosion. As a result, the surfactant inhibits MS corrosion, confirming the results of the weight loss approach [73]. In polarization curves (figure 5d), SDS is a pickling-type inhibitor that inhibits the corrosion without altering *E*_corr_ values [74].

#### 3.2.3. Variation of corrosion potential versus concentration of surfactants

Polarization measurements of surfactant SDS in a 0.5 M H_2_SO_4_ solution in the MS sample are illustrated figure 5b,d, where differences in their rates of corrosion potential can be seen. The corrosion potential rates decreased with an increase in the surfactant concentration. As the surfactant content increases, the corrosion rates decrease and hence, the corrosion potential also decreases. As a result of surfactant adsorption, corrosion rates are reduced.

#### 3.2.4. Variation of inhibition efficiency (%) versus sodium dodecyl sulfate concentration in 0.5 M H_2_SO_4_

A higher percentage of inhibition efficiency signifies a greater effectiveness of the corrosion inhibitor in reducing corrosion. The correlation between inhibitor inhibition efficiency and its effectiveness in the corrosion field is illustrated in figure 5c,d. Potentiodynamic experiments reveal that an increase in the number of surfactants leads to an increase in the presence of inhibitor molecules, i.e. SDS is available for adsorption on the MS surface [75]. Consequently, this reduces the exposed surface area that is susceptible to a direct acid reaction on the metal surface. The inhibition efficiency of SDS at concentrations of 0.0019 M, 0.0034 M, 0.0077 M and 0.0154 M is reported as 85.77, 93.05, 96.01 and 96.01%, respectively. This underscores the effectiveness of surfactant SDS as a robust inhibitor of MS. In particular, SDS shows a high efficiency of nearly 98.29%, indicating its substantial inhibitory capabilities. These findings align with the data from the weight loss method, further supporting the conclusion that SDS is an effective inhibitor for MS.

### 3.3. Fourier transform infrared analysis

To study the chemical identity, purity and molecular interactions within the sample, FTIR analysis of the SDS-coated MS sample was carried out in the wavenumber range of 500–4000 cm⁻¹.

FTIR spectra were used to study the protective coating that forms on metal surfaces. The goal was to understand how the organic inhibitor, which attaches to the metal surface, bonds with it [76]. Figure 4a illustrates that a protective layer was formed on the metal surface after 9 h immersion in 0.0077 M SDS solution. Interestingly, there is a noticeable change in the stretching of phenolic OH from 3805 cm⁻¹ to 3408 cm⁻¹. Furthermore, the stretching frequency of the methyl group’s C-H bond has shifted from 2840 to 3000 cm⁻¹ [77].

**Figure 6.**
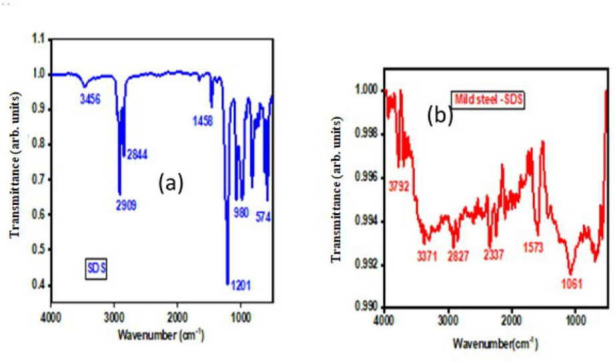
(a) FTIR diagram of sodium dodecyl sulfate (SDS). (b) FTIR diagram of SDS–mild steel. FTIR, Fourier transform infrared spectroscopy.

Moreover, there has been a decrease in the stretching frequency of the C-O bond from 1338 to 1020 cm⁻¹. Similarly, the stretching frequency of the S=O bond of the sulfate group has changed from 1415 to 1330 cm⁻¹ [78]. In figure 6b, the FTIR spectrum shows the protective coating that developed on the fragment after a 9 h soak in 0.0077 M SDS solution. There is a change in the stretching of the phenolic -OH group, shifting from 3805 to 3378 cm⁻¹ [79]. The stretching frequency of the C-H bond in the alkane group has slightly increased from 1370 to 1365 cm⁻¹, while the stretching frequency of the C-O bond has slightly decreased from 1338 to 1335 cm⁻¹. This suggests that the protective layer comprises a metal–SDS complex.

### 3.4. Field electron scanning electron microscope and X-ray energy dispersive spectroscopy

**Figure 7.**
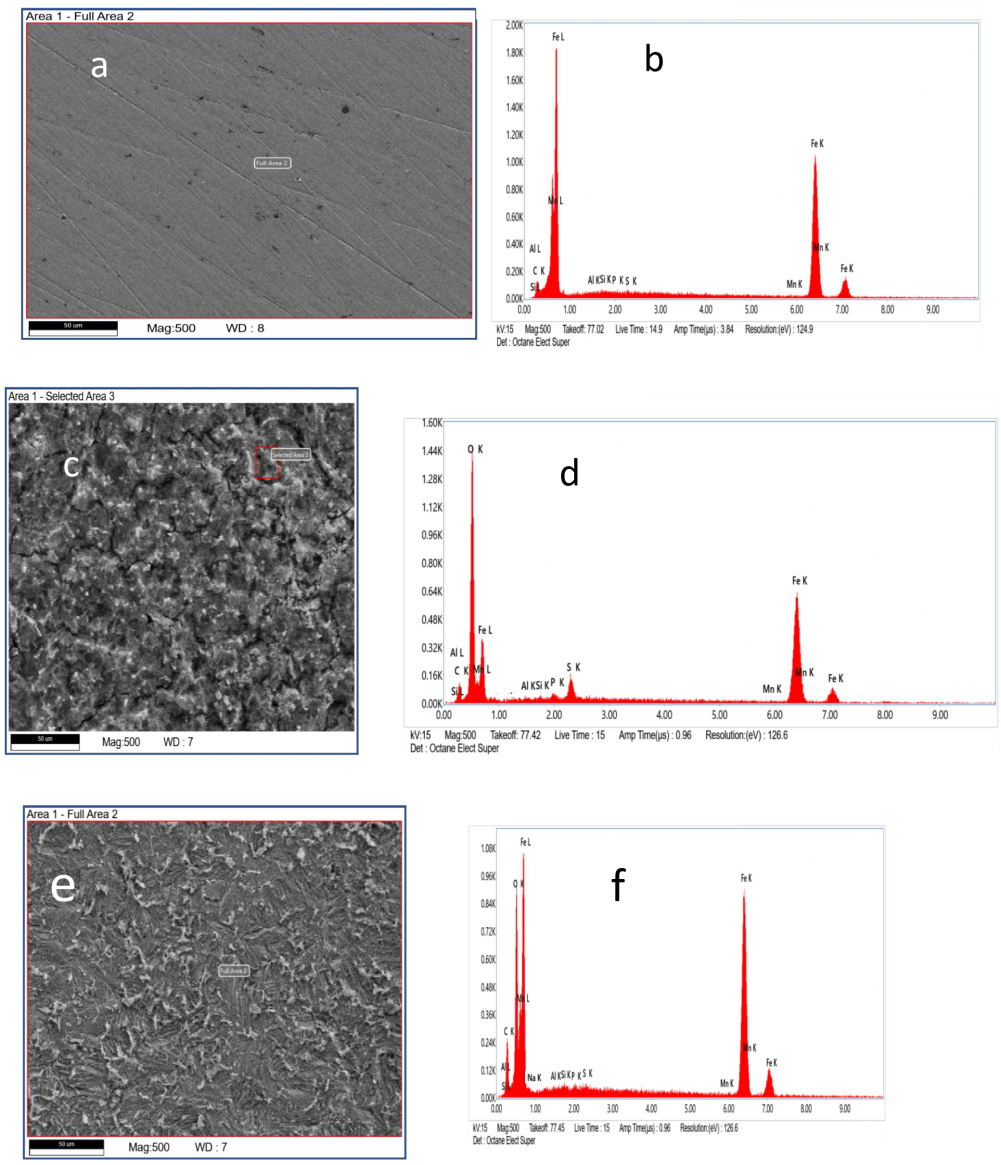
(a) Surface of bare mild steel. (b) Picture of bare mild steel from EDX. (c) The mild steel surface in 0.5M H_2_SO_4_ after 9 h without SDS. (d) The EDX image of mild steel after 9 h in 0.5M H_2_SO_4_ in the absence of SDS. (e) Mild steel at CMC after 9 h in 0.5 M H_2_SO_4_ with SDS present. (f) Mild steel’s EDX picture at CMC after 9 h in 0.5 M H_2_SO_4_ with SDS present. EDX, X-ray energy dispersive spectroscopy; SDS, sodium dodecyl sulfate; CMC, critical micelle concentration.

FESEM analysis was used to examine the surface morphology of MS specimens before and after corrosion inhibition. Figure 7a,b show the FESEM and EDX images of the bare MS before dipping it into a sulfuric acid solution. The surfaces of the polished steel samples were uniformly smooth. Due to a rapid breakdown process, the bare surface exhibits a highly porous structure with deep and wide fissures, mainly composed of iron on MS. Figure 7c shows the FESEM image of the MS sample after it was immersed in 0.5 M H_2_SO_4_ solution. From this, it is clear that H_2_SO_4_ has a detrimental effect on MS that leads to significant corrosion. Figure 5d shows the FESEM image of the sample in the presence of SDS. The surface of MS corrodes far less in the presence of inhibitors (SDS) than in the blank solution, as seen in figure 7c [80]. After 9 h of immersion in SDS, the inhibitor molecules form an adsorbed layer on the MS surface. The formation of such a layer by SDS molecules contributes to the protection of the surface of the MS by shielding it from corrosive species in the solution [81]. Using an EDX analysis, the relative amounts of each element on the surface of MS were determined. SDS reduces the amount of iron in a 0.5 M H_2_SO_4_ solution while increasing the amounts of sulfur, carbon and oxygen on the surface of MS. This study shows that SDS adsorption forms a shielding layer on the surface of MS surface that inhibits the dissolution of iron [82]. EDX measurements of element percentage are given in table 1.

**Table 1.** Elemental composition analysis of mild steel in different conditions using SEM/EDX analysis. SEM, scanning electron microscopy; EDX, X-ray energy dispersive spectroscopy.

condition of mild steel	Wt %		
	Fe K	C K	O K
bare condition	85.7	13.2	—
immersed in 0.5 H_2_SO_4_ without SDS	49.5	14.8	32.1
immersed in 0.5 H_2_SO_4_ with SDS at CMC	63.0	19.7	16.3

Hence SEM EDX analysis illustrated the decrease in iron atom from 85.7 to 49.5 % with the increase in oxygen atom to 32.1% when MS is immersed in 0.5 M H_2_SO_4_ due to the formation of the corrosion-related products on the surface of MS immersed, but the Fe atom is 63.0% with 16.3% O atom when it was immersed for 9 h in H_2_SO_4_ with SDS. This validated SEM image shows that SDS as surfactants reduce the corrosion process preventing the dissolution of iron in MS surfaces (table 1; electronic supplementary material, tables S3–S5).

### 3.5. Atomic force microscopy study

**Figure 8.**
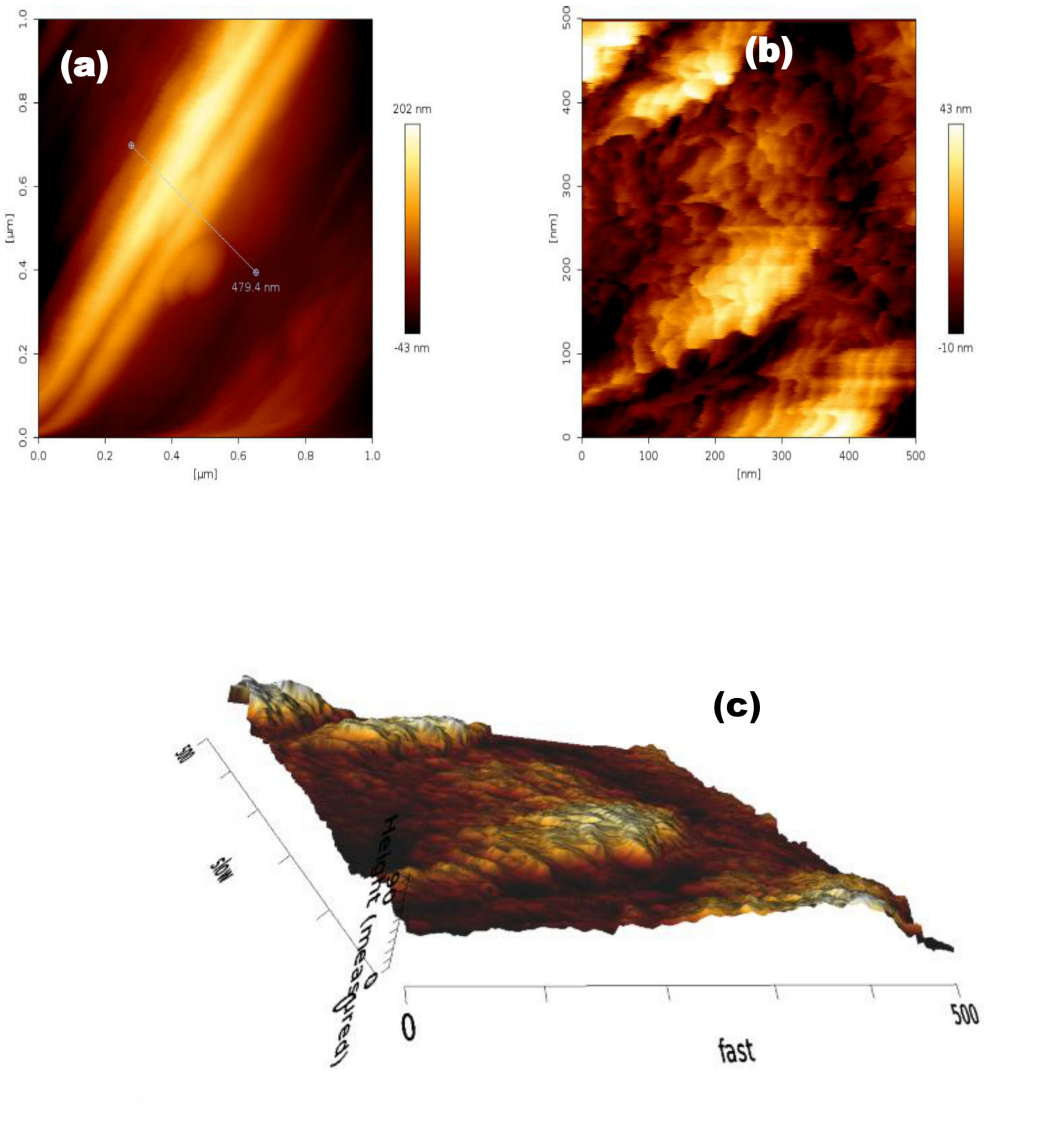
(a) The surface of mild steel after 9 h in 0.5M H_2_SO_4_. (b) Mild steel after 9 h in 0.5 M H_2_SO_4_ in the presence of SDS. (c) Three-dimensional mild steel after 9 h in 0.5 M H_2_SO_4_ in the presence of SDS.

Morphological changes on the surface of MS were analysed using AFM, with and without inhibitors. Figure 8a shows the AFM images of MS that were polished, sonicated [83], washed and dried and then it was immersed in an acidic solution of 0.5 M H_2_SO_4_. Figure 8b shows pictures of MS in the SDS solution, the acid solution of 0.05 M H_2_SO_4_, and figure 8c shows the three-dimensional picture of MS in the SDS solution.

However, inhibitors significantly reduce surface roughness and improve the protective barrier of MS against SO_4_^−2^ ion corrosion (seen in figure 8a), resulting in fewer high points and shallow depressions. Additionally, the surface roughness of the steel samples can be calculated using the height profiles obtained from the AFM examination (as shown in figure 8b). The root mean square (RMS) roughness of the polished steel samples is presented. However, after 9 h of exposure to 0.5 M H_2_SO_4_, the surface had significantly deteriorated and showed RMS roughness. The presence of SDS causes the RMS to drop to 98 nm, indicating the efficacy of the inhibitors [80].

After being immersed in 0.5 M H_2_SO_4_ solutions, figure 8 shows the morphologies of the MS samples with and without SDS. The surface showed extremely uneven topography as a result of corrosion attack in the absence of the inhibitor (figure 8a). According to figure 8a, the average roughness of MS in 0.5 M H_2_SO_4_ solution without inhibitor was −43 nm to 202 nm. The shielding inhibitor layer that formed on the surface of the MS when there was SDS reduced corrosion damage and produced a smoother surface (figure 8b). Without an inhibitor, the MS surface was extensively corroded (figure 8a). On the other hand, the inhibitors caused the surface of the sample to become smoother (figure 8b) [84]. This is because inhibitor molecules bind to the reaction sites on the MS surface and exhibit a consistently strong inhibitory effect, which reduces the roughness of MS from −10 nm to 43 nm. The amount of interaction between the MS and SDS was shown by AFM test.

Figure 8c shows the three-dimensional image of the MS in the SDS solution. Surface roughness (*R*_a_) values obtained from three-dimensional-AFM topographic image analysis are 90.0 nm for MS immersed in 0.5 M H_2_SO_4_ with SDS. This indicates the decrease in surface roughness of immersed MS specimens with SDS that behave as surfactants with anticorrosion behaviour. When SDS is added to acid solution that protects the surface of MS from corrosion which is also validated with the peak to valley roughness values (*R*_t_) which is reduced by about 44.16% from 320.9 nm to 179.2 nm. These results align with the results obtained from SEM morphological analysis.

### 3.6. Mechanism of inhibition in mild steel

The prevention of metal corrosion using SDS can be ascribed to the adsorption of large molecules onto the metal surface, effectively hindering active corrosion sites. The effectiveness of this inhibition is influenced by the functional and amphiphilic characteristics of the chemicals involved [66]. As suggested in previous studies, the removal of water dipoles from a metal surface enhances the binding of inhibitor molecules [61].


$$MS+water+H_{2}SO_{4}=MS-acidic\;water$$

MS+SDS(sol)inacidicwater=MS−SDS(ads)complex

**Figure 9.**
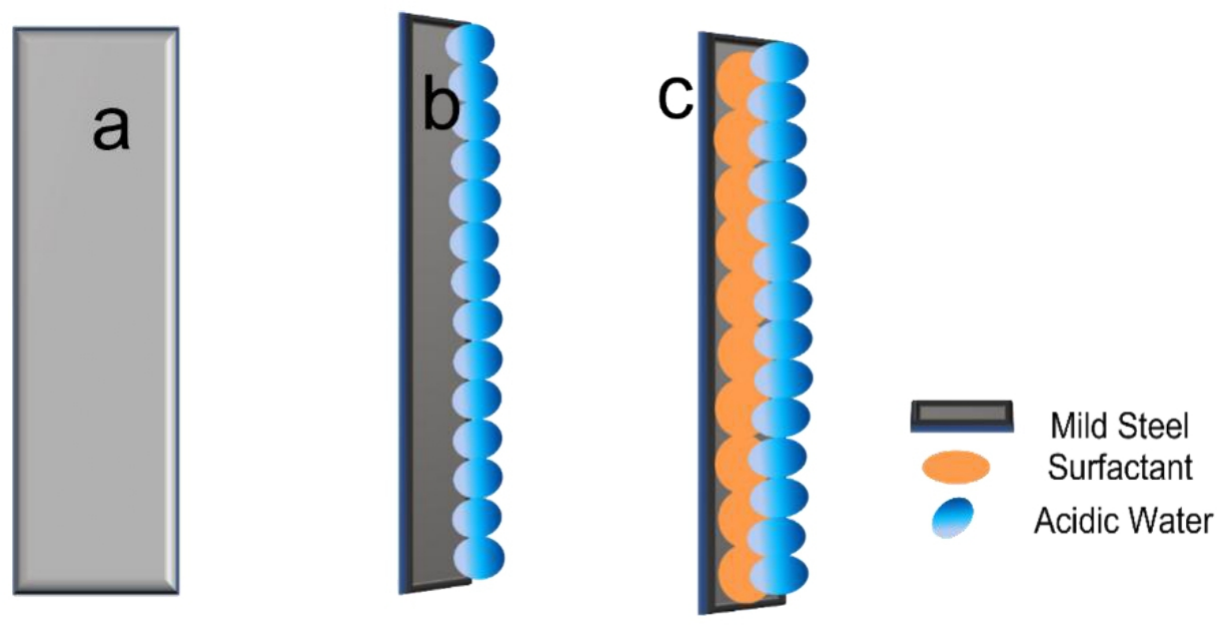
(a) Polished mild steel. (b) Water molecule adsorbed on the surface of the polished mild steel surface. (c) Sodium dodecyl sulfate adsorbed on the surface of the mild steel.

Figure 9a displays the polished MS with its glass surface that resembles a mirror. Figure 9b shows that water molecules are adsorbed on the polished MS surface. In figure 9c it can be seen that SDS adsorbed and formed a thin barrier layer on the surface of MS. By shielding the metal from contact with acid or water, this barrier lessens the amount of corrosion. [85]. As opposed to SDS (sol), SDS (ads) refers to organic molecules that are adsorbed to metallic surfaces. H_2_O (ads) denotes water molecules adsorbed on the metal surface, and the size ratio indicates how many water molecules are replaced by one SDS molecule. After treatment with SDS, MS shows an open circuit potential (OCP) of −0.47 V. Potential zero charges of MS were found to be negative for the OCP value in the sulfate solution. An OCP value higher than the potential zero current can induce a positive charge on the metal surface. Consequently, in an acidic solution, inhibitor molecules, having acquired a positive charge through the abstraction of protons by a single pair of oxygen and sulfur, may electrostatically adsorb onto a negatively charged metal surface due to SO^2−.^ In an acidic environment, SDS, containing a sulfate group, can undergo deprotonation, resulting in a negative charge [76].

**Figure 10.**
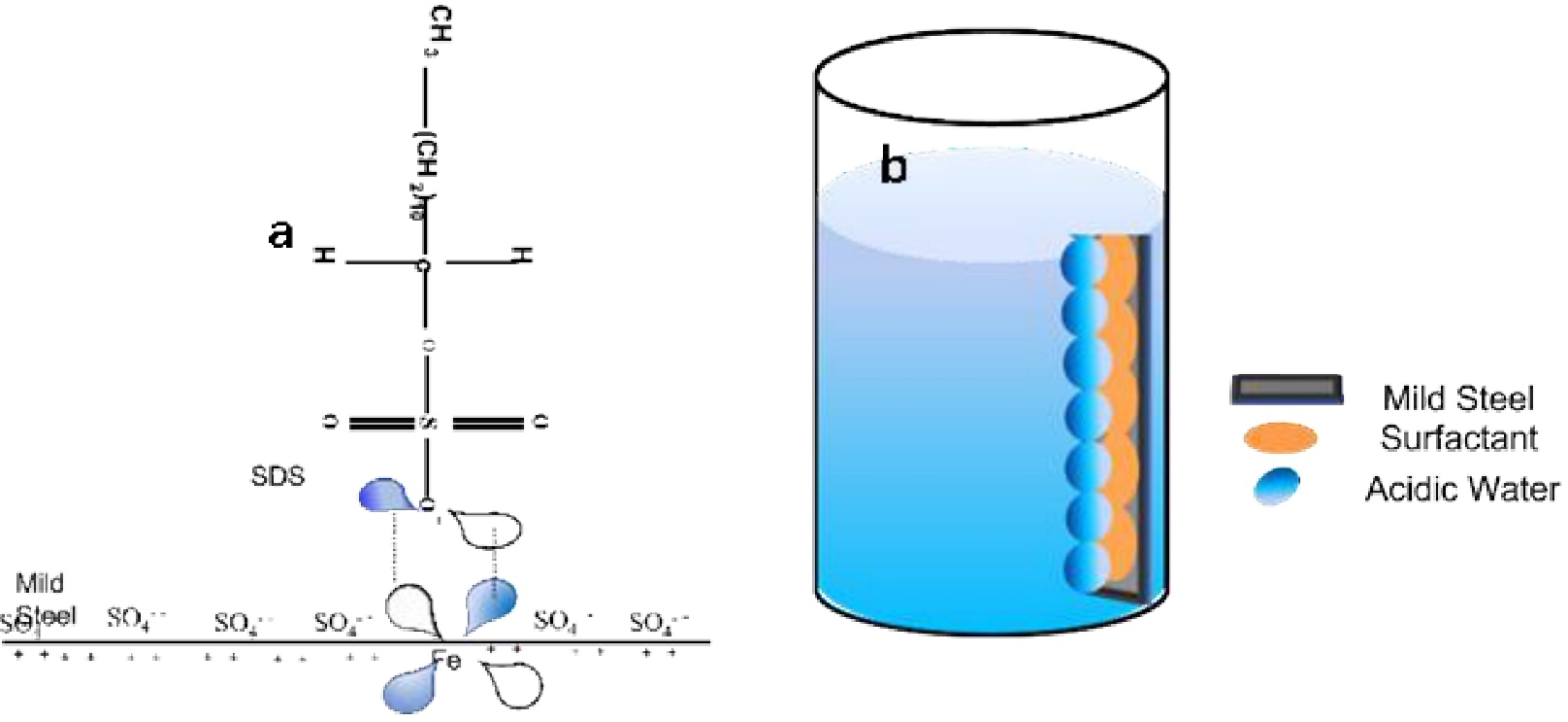
(a) Mild steel surface and sodium dodecyl sulfate (SDS) electrons donor–acceptor interaction. (b) Mild steel immersed in surfactant solution.

Sulfate radicals can be adsorbed on a MS surface with negatively charged surfaces, inducing a negative charge on the metal [9]. Negatively charged SDS molecules can adsorb by electrostatically attaching to the sulfate ion. In an acidic solution, positively charged SDS may compete with H^+^ ions on the MS surface for electrons. On the other hand, SDS returns to its neutral form when H_2_ molecules are released. The vacant iron d orbital in MS can then engage with the highest occupied molecular orbital (HOMO) of the SDS molecule to form a coordinating bond (donor–acceptor) [86]. The p-orbital bonding, illustrated in figure 10, which exhibits the highest electron density in the SDS molecule, can function as a HOMO [87].

The donor–acceptor interaction gives the MS surface an excess negative charge that could push iron’s electrons back into the lowest unoccupied molecular orbital (LUMO). The LUMO corresponds to the vacant orbital p* (antibonding) of the inhibitor molecule. In the SDS molecule, the antibonding orbital p* (as shown in figure 10) exhibits a larger orbital size. Figure 10 illustrates a fundamental inhibitory process involving a molecule of SDS on the MS sample [61]. It is important to note that the molecular interactions depicted are illustrative rather than based on precise theoretical calculations. The corrosion potential (*E*_corr_) was the electrochemical parameter that was significantly altered by the addition of SDS surfactants. MS had the lowest corrosion potential in 0.5 M H_2_SO_4_ without addition of SDS, measuring −0.4719 V. With the addition of SDS, an extra adsorbed layer of SDS can be formed on the MS’s inherent protective oxide layer. The proportion of the surfactant layer coverage on the surface increases before reaching CMC as the quantity of SDS increases [63].

When the natural oxide is removed and an additional protective layer is introduced with a surfactant, the initiation of corrosion on the surface of MS becomes more challenging for hostile media. Consequently, the corrosion potential increases to a higher level, extending the passive range [88]. In the presence of SDS in the solution, corrosion requires a stronger oxidizing potential. When the CMC is reached, the surfactant layer envelops the MS surface thoroughly after a secondary passivation stage. Beyond the CMC concentration, no corrosion is observed on MS samples [73]. The complete prevention of corrosion in surfactant-containing solutions at and beyond the CMC is attributed to the corrosion potential that exists in the oxygen evolution region. Elevating the corrosion potential to the level of oxygen evolution demonstrates the ability of the surfactant to prevent full corrosion [87].

The anodic segments of the polarization curves, generated by solutions containing SDS surfactant at CMC and beyond, exhibit virtual uniformity, particularly at high anodic overvoltage.

However, increasing the SDS surfactant concentration beyond the CMC level does not impact the passive behaviour of MS. This underscores the importance of CMC as the optimal anionic surfactant concentration to mitigate MS corrosion in 0.5 M H_2_SO_4_ [89]. In figure 5d, the polarization curve for the solution in the absence of SDS shows a potential of −0.4719 V, significantly higher than the corrosion potential. On the contrary, the SDS solution at CMC maintains a passive range with a potential of −0.4793 V. In the absence of SDS, corrosion is evident on the MS surface post-measurement. With SDS at CMC, the corrosion current of MS remains constant for the equivalent potential and oxidizing power.

## 4. Conclusions

(i) Surfactants are essential to reduce the corrosive current of MS and preserve uniformity after CMC.
(ii) The CMC for SDS in 0.5 M H_2_SO_4_ is determined to be 0.0077 M by conductivity measurement. In 0.0077 M of SDS surfactant solution, it demonstrates an inhibitory efficiency of more than 96% in 9 h, demonstrating a remarkably high effectiveness at 298.15 K with an IE of 96.01%.
(iii) The inhibitory efficiency exhibits an upward trend with increasing SDS concentration, reaching a maximum peak in a solution of 0.0077 M. This trend observed is consistent with the potentiodynamic measurement approach.
(iv) The inhibitor may act as a mixed-type inhibitor, on the basis of the minimal drop in OCP. A protective layer made of deposited SDS molecules is formed, impeding the hydrogen reduction reaction, as seen by consistent cathodic Tafel slopes with increasing SDS content.
(v) FTIR spectrum analysis reveals that the protective deposit is a mixture of metal and SDS. The examination of the surfaces using EDX, FESEM and AFM verify that the MS surface has a protective SDS layer that effectively prevents corrosion. Therefore, SDS effectively functions as a surfactant inhibitor for MS in acidic conditions, increasing the durability of MS life and avoiding corrosion.

## Data Availability

Data that support this study have been uploaded as electronic supplementary material [90].
